# Edible evolution: the significance of food additives in shaping human health

**DOI:** 10.3389/fnut.2025.1717352

**Published:** 2026-01-08

**Authors:** Avery Erickson, Shreya Bellampalli, Arthur Beyder

**Affiliations:** 1 Idaho College of Osteopathic Medicine, Meridian, ID, United States; 2 Enteric Neuroscience Program (ENSP), Division of Gastroenterology & Hepatology, Mayo Clinic, Rochester, MN, United States; 3 Medical Scientist Training Program, Mayo Clinic Alix School of Medicine, Mayo Clinic, Rochester, MN, United States; 4 Division of Gastroenterology and Hepatology, Department of Medicine, Mayo Clinic, Rochester, MN, United States; 5 Department of Physiology and Biomedical Engineering, Mayo Clinic, Rochester, MN, United States

**Keywords:** gut health, gut microbiome, nutrigenomics, nutritional anthropology, nutritional ecology

## Abstract

Human diets have transitioned through distinct stages, from foraging to agriculture, domestication, and industrialization, that progressively altered food composition, availability, and ecological context. While these shifts enhanced food security and shelf life, they also narrowed dietary diversity and layered non-nutritive components ranging from salt and fermentation by-products to synthetic dyes, preservatives, artificial flavors, and non-caloric sweeteners onto ancestral nutritional frameworks. This review traces the historical integration of such compounds, situating them within broader dietary transitions to highlight how technological innovations gradually distanced human diets from their evolutionary origins. Drawing on nutritional anthropology, archaeogenomics, and ecological perspectives, we examine how these changes reshaped gut health, microbial diversity, and long-term disease risk. By framing non-nutritive additives within the long arc of dietary evolution, this perspective provides context for understanding the rise of modern, ultra-processed food systems and underscores the importance of integrating evolutionary and ecological evidence into contemporary nutrition and gastrointestinal research.

## Introduction

**Figure 1:**
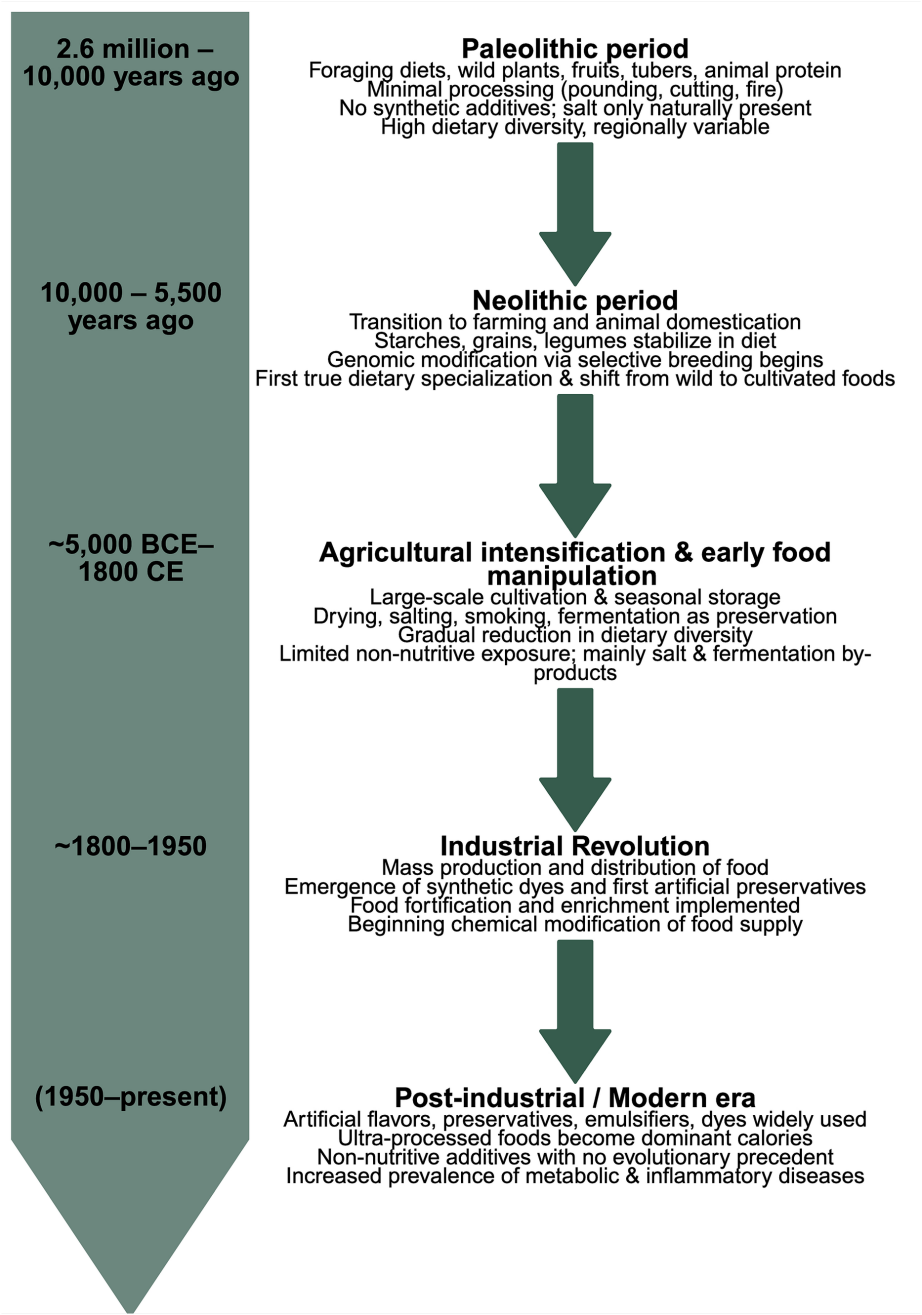
Timeline of human diet evolution. This timeline illustrates major transitions in dietary composition across human history, including ecological foraging-based diets (Paleolithic), agricultural reliance and plant/animal domestication (Neolithic-early agrarian), early food processing and genomic modification, the introduction of synthetic additives during industrialization, and the emergence of ultra-processed food systems in the modern era. Each period is annotated with indicative food sources, processing methods, and typical non-nutritive exposures.

This review aims to highlight the role of non-nutritive dietary components–historically overlooked in nutrition research–and their emerging significance in modulating gut health. Assessing how diet influences human physiology and gut health requires perspectives that extend beyond modern nutrition science. Comparative evidence from nutritional anthropology, archaeogenomics, and evolutionary biology illustrates how dietary practices, from early hominids to contemporary populations, have shaped metabolism, supported physiological function, and influenced long-term health trajectories (1, 2). While early human diets were shaped primarily by ecological availability, they involved minimal use of additives beyond naturally occurring salts, fermentation, or smoking techniques for preservation. In contrast, the modern food supply is increasingly characterized by non-nutritive compounds such as synthetic dyes, preservatives, and artificial flavors that have no precedent in evolutionary history (2). We recognize non-nutritive dietary compounds as non-caloric and not essential for human health and survival. Tracing this trajectory from prehistoric reliance on low-processed foods to today’s additive-rich food environment provides critical context for understanding diet-related disease burdens such as metabolic syndrome, cardiovascular diseases, and cancer (Figure 1).

The aim of this review is to synthesize and evaluate evidence on how non-nutritive components of the human diet, including preservatives, dyes, synthetic sweeteners, and other additives, have emerged across dietary history and now influence gut health and chronic disease risk. We aim to situate these exposures within an evolutionary context, comparing modern diets to ancestral patterns, and to identify areas for mechanistic hypotheses that warrant further investigation. We intended for this framework to support both scientific inquiry and future nutritional guidance.

We completed the review of literature using PubMed and Google Scholar using keyword searches to identify relevant literature, and publication dates to ensure inclusion of timely and appropriate articles. Therefore, we excluded articles published prior to the year 2000, and as a result the majority of included articles were published on or after 2010.

A growing body of evidence demonstrates that non-nutritive dietary components exert biological effects beyond their intended technological function. For example, preservatives like benzoates and nitrites extend shelf life but may also influence systemic inflammation and the gut microbiome (3, 4). Artificial flavors and colors enhance palatability and sensory appeal, yet emerging studies suggest they can alter microbial communities or contribute to intestinal irritation (5). Similarly, non-nutritive sweeteners, sugar alcohols, and novel compounds such as allulose modify glycemic responses but have also been implicated in changes to satiety signaling and metabolic risk (6). These categories illustrate that while such compounds provide functional benefits to food systems with regard to palatability and convenience, their widespread and often under-regulated use introduces exposures with uncertain long-term physiological consequences.

The high energy density of the modern diet and increased intake of food additives may be a major contributor to the development and progression of chronic diseases that are pervasive throughout the global population (7). Reconstructing ancestral diets and identifying adaptive responses to various food environments can help explain the emergence of modern metabolic and inflammatory disorders.

Gut health–a multifaceted concept encompassing gastrointestinal physiology, intestinal barrier integrity, and the gut microbiome–has become central to understanding the diet-disease relationship (8). In particular, alterations in gut health have been implicated in the etiology of several chronic conditions, including colorectal cancer, cardiovascular disease, and arthritis (9).

## Historical evolution of the human diet

### Pre-industrial diet and diet plasticity

Throughout most of evolutionary history, the foundation of the human diet was based on regional-specific foods, providing the majority of food consumed. This variety was influenced not only by regional availability but seasonal availability and wild game presence as well. Among early primates in Africa and surrounding regions, plants such as grasses, seeds, tubers, and fruits were the core of the diet, while animal foods including birds, eggs, reptiles and others served as supplementary sources (2). This speaks to a concept known as diet plasticity, whereby an organism has the ability to adjust its diet in response to changes in food availability, quality, or environmental conditions (10). Into the Pleistocene period (from 2.6 million to 10,000 years ago) (11), meat, starch-containing carbohydrates, and easily digestible carbohydrates became more of a mainstay in the diet due to climate change, dentition adaptations, and the emergence of cooking/food preparation techniques (2, 12). Pleistocenic dietary changes were also critical for the increasing brain size and energy requirements of early hominids (2).

The emergence of simple cooking techniques–including the use of fire and mechanical processing–allowed hominids to increase the energetic yield, meaning the usable calories per pound, of meat and plant foods and decrease necessary chewing. Mechanical techniques included pounding and grinding of foods to tenderize them and the use of stone flakes to cut food into smaller pieces as well as remove skins, hides, and cartilage (12). The use of fire in food preparation facilitated gains in energy yield of difficult-to-digest foods, such as complex carbohydrates and meat. This technique also played a key role in eliminating harmful pathogens. While fire and mechanical processing improved the energetic yield from foods, humans needed a way to optimize the stability and long-term safety of the foods. The introduction of salt, what we now know as table salt or NaCl, one of our earliest recognized non-nutritive additives, also provided the ability to not only safely preserve foods without refrigeration but also supply essential minerals necessary for fluid balance, nerve function, and muscle contractions (13).

Despite their diversity, pre-industrial diets did not always effectively prevent nutrient deficiency. Nutrient deficiencies have only been widely recognized and investigated in recent human history, gaining traction in the late 19th to early 20th centuries. There is some evidence of anemia and low bone mineral density secondary to suspected iron deficiency, low B12 intake, or low vitamin D in humans of the Neolithic period (9000–5,500 years ago) (14), following the Paleolithic period (2.6 million to 10,000 years ago) (15), but further rigorous investigation is needed to elucidate the etiology. In the 19th century, Eijkman discovered that chickens fed polished rice developed polyneuritis, identical to beri-beri. The polished rice lacked the thiamine-containing bran/germ layer seen in unpolished rice. Similar observations were later observed in humans, highlighting the effects of nutrient deficiency (16). In the early 20th century, Blegvad discovered that vitamin A supplementation in kids could treat xerophthalmia and keratomalacia (17). Eijkman and Belvad’s discoveries likely helped pave the way for future fortification and enrichment of foods. While the addition of vitamins to food provides essential micronutrients and does not meet non-nutritive additive criteria, they likely facilitated increased public acceptance of food additives in general, allowing future non-nutritive additive use. Nutrient deficiency is still a modern occurrence, especially among populations who consume an energy-dense, nutrient-poor diet or those who face food insecurity (18).

### Food domestication and agricultural intensification

The Neolithic Revolution initiated large-scale food production and the domestication of plants and animals. The domestication of food has not only contributed to improved food security by increasing the amount of food produced, but also manipulated the genomes of foods to alter palatability, macro-, and micronutrient content. Genomic interventions by humans have also increased the number of harvests and the mass of food gathered per harvest (19). For example, apples have not only become larger, sweeter, firmer, but they are also more resistant to disease through specific single-nucleotide polymorphisms (20). A wide variety of plants have been genetically modified to be resistant to certain diseases and, notably, pesticides as well (21). Non-domesticated ancestral foods were found to contain higher amounts of xenobiotics, or foreign compounds, such as heavy metals like arsenic and mercury. Xenobiotics can lead to alterations in the gut microbiome (19, 22). For example, arsenic has demonstrated the ability to decrease *Bacillota* phylum in the gut of mice (23) and mercury exposure in animal models leads to survival and proliferation of mercury-resistant bacterial strains in the gut, such as *Pseudomonas* and *Listeria* (24). Animals were also domesticated, which largely replaced hunting, to provide meat and dairy products (25). Food domestication and genetic modification of foods addressed food availability and food security, allowing the focus to shift from whether there was enough food to whether the food tasted good.

### Industrialization and processed foods

In the 18th century, the Industrial Revolution transformed food systems through mass production, transportation, and synthetic ingredient development. Unlike the pre-industrial diet, the post-industrial diet utilizes different cooking techniques and fosters an environment where convenient, “fast-foods” are widely consumed and preferred (26). Food fortification is defined as adding micronutrients that were not previously present to commonly consumed foods, such as breads and cereals, to increase their overall nutritional value. Fortification and enrichment of foods has been implemented to combat nutritional deficiencies in developed countries since the early 20th century, starting with iodized salt, and this practice is spreading to low- and middle-income countries around the world (27, 28). Enrichment of food is described as adding back nutrients previously lost during processing. Most commonly, this is seen in grain products (29). The practice of enrichment was born out of necessity during the early 20th century to address widespread micronutrient deficiencies in the general population. Micronutrient deficiencies became particularly visible when evaluating military recruits for service during the First World War, where conditions such as iron-deficiency anemia, goiter, and beriberi were prevalent (30, 31). Improving the nutrient content of staple foods could help alleviate population-level deficiencies without requiring major dietary shifts. Enrichment of wheat flour with B vitamins and iron, alongside fortification strategies such as the addition of iodine to salt, represented some of the earliest systematic nutrition interventions. Nutrient enrichment programs significantly reduced deficiency-related conditions, such as rickets and pellagra, across diverse populations (32). Today, enrichment and fortification remain cornerstones of food processing worldwide, with fortified grains, iodized salt, and vitamin D-supplemented milk continuing to provide broad nutritional benefits, particularly in populations with limited dietary diversity. Despite their success, questions remain about balancing the benefits of fortification with potential unintended consequences, such as excessive intake or dependence on highly processed foods as nutrient delivery vehicles (28).

Non-nutritive food additives, such as synthetic food dyes and preservatives, began appearing in the modern food supply in the mid-late 19th century and are a hallmark of the post-industrial diet. Synthetic food dyes were added to foods to enhance the appearance of foods or restore sensory qualities lost during processing. Synthetic food dyes are commonly synthesized from coal tar derivatives and potentially pose some risk to human health, such as gut microbiome changes and potential colonic inflammation (33). Synthetic dyes have the potential to be overused and pose a challenge to federal regulation (34). Food preservation techniques have also been adapted from our early human ancestors. Modern chemical preservation techniques for food, such as benzoates, sorbates, and nitrites, provide the opportunity to preserve foods in different ways compared with ancient techniques, such as vinegar, salt, and smoking, which are still also used today. Modern, post-industrial preservatives are used depending on the composition of food to be preserved, the type of spoilage, and the desired shelf-life of the food (35). For context, sodium benzoate specifically is reported to extend shelf life of certain foods from about 6–10 weeks without preservatives to at least 20 weeks with 0.1% sodium benzoate, showing an extension roughly 2 to 3 times longer or more depending on packaging and storage. Pre-industrial foods, lacking such chemical preservatives, relied mostly on natural methods (drying, salting, fermenting) that extended shelf life variably but likely far less reliably or for shorter periods than modern preservatives allow. Hence, we hypothesize that the addition of sodium benzoate and similar preservatives have extended shelf life from weeks/months in pre-industrial times to several months or years in many modern processed foods, effectively adding up to a few years in preserved shelf life compared to pre-industrial foods (36, 37). Chemical preservatives present an opportunity for changes to human health, such as potential changes to systemic inflammation and gut microbiome changes (38, 39). Artificial flavors were added to foods to enhance or add additional flavors to foods and improve palatability. Use of artificial flavors increased significantly when the culture of food began to emphasize packaged foods and eating outside of the home more often (40). In the late 20th century, added sugars were introduced to enhance flavor, and became a mainstay in the Western diet when eating priorities shifted toward convenience and cost-effectiveness. This new diet trend coincided with the rise of obesity and other metabolic disease rates (41). From 1990 to 2021, the global prevalence of adult obesity increased dramatically: by about 155% in males and 105% in females. In 1990, obesity rates were much lower, and by 2021 an estimated 1 billion men and 1.11 billion women worldwide were overweight or obese. This represents more than a doubling in obesity prevalence globally over roughly three decades (42–44). For this purpose, non-nutritive sugars such as allulose and sugar alcohols such as xylitol and erythritol were introduced to allow a decrease in caloric intake while still maintaining taste (29, 45, 46). Non-nutritive sweeteners also exert implications on the development of metabolic syndrome (6).

We hypothesize that as is evident from the manipulation of food with artificial additives, food processing is recognized as a hallmark of the post-industrial and modern diet. As the focus shifted toward palatability and convenience, grocery stores saw the introduction of “ultra-processed” and “processed” foods. Processed foods with extensive addition of non-nutritive compounds including preservatives, emulsifiers, flavorings, and colorings, can have differing levels of intervention, obtaining designations from “minimally processed” up to “ultra-processed” foods. A diet high in processed foods leads to an increase in calories, carbohydrate, and fat intake, ultimately leading to weight gain (47). Controlled feeding studies provide strong causal evidence for this relationship. In a landmark randomized controlled trial, Hall et al. in 2019 demonstrated that individuals consuming an ultra-processed diet ad libitum ingested ~500 more kilocalories per day and experienced significant weight gain compared with those consuming a minimally processed diet, despite matched macronutrient composition and palatability. Beyond excess energy intake, large-scale epidemiological studies have linked ultra-processed food consumption to higher risks of obesity, cardiovascular disease, and all-cause mortality. Srour et al. (48) reviewed this body of evidence, highlighting both epidemiological associations and emerging mechanistic insights, including impacts on satiety signaling, glycemic control, systemic inflammation, and gut microbiome composition. Together, these findings underscore that diets dominated by ultra-processed foods not only promote weight gain but also elevate the risk of chronic systemic disease, positioning them as a major contributor to the global burden of non-communicable diseases. In specific, non-communicable diseases indicate medical conditions that are not infectious and cannot be transmitted from one person to another. They are typically chronic, meaning they last for a long time and generally progress slowly. Examples include cardiovascular diseases (such as heart attacks and strokes), cancers, and diabetes (49).

## Discussion

The incorporation of non-nutritive components into the human diet represents one of the most striking shifts in dietary history. From early reliance on naturally occurring salts, fermentation, and smoking, to the widespread use of synthetic preservatives, dyes, flavors, and sweeteners, each stage of human development has added layers of compounds that extend beyond caloric or nutrient value. This trajectory reflects not only advances in food preservation and palatability but also the progressive distancing of human diets from their ecological and evolutionary roots.

In the pre-industrial era, non-nutritive additions were limited, often arising from necessity–such as salt for preservation or fermentation for safety and digestibility. With agricultural intensification, domestication and early food processing began altering the chemical composition of foods, yet the diet remained anchored in relatively unmodified plant and animal products. During industrialization (Table 1), the pace and scale of dietary change accelerated. The 19th and 20th centuries introduced synthetic dyes, chemical preservatives, artificial flavors, and non-caloric sweeteners into the global food supply. These compounds were designed to improve shelf life, enhance sensory appeal, and lower costs, but in doing so, they fundamentally changed both the composition of the human diet and the nature of human exposures.

**Table 1:** Comparison of key features in pre-industrial and post-industrial diets.

Feature	Pre-industrial diet	Post-industrial diet
Primary food sources	Wild plants, tubers, fruits, seeds, and game meat, with regional/seasonal variability	Mass-produced, processed, and packaged foods, often shelf-stable and widely distributed
Processing techniques	Mechanical (pounding, grinding, cutting), use of fire for cooking and pathogen reduction	Industrial food processing, refining, chemical stabilization, large-scale manufacturing
Non-nutritive additives	Naturally occurring: salt for preservation, smoke curing, fermentation by-products (acids, alcohols)	Synthetic preservatives (benzoates, sorbates, nitrites), synthetic dyes (coal tar derivatives), artificial flavors, emulsifiers
Purpose of additives	Preserve food safety without refrigeration, enhance digestibility, provide essential minerals	Extend shelf life, restore/enhance sensory qualities, improve palatability, lower production costs, enhance convenience
Impact on nutrient profile	Minimal nutrient loss; nutrient density largely preserved; occasional deficiencies due to ecological constraints	Nutrient enrichment/fortification (iodine, iron, vitamins) counterbalanced by dilution of nutrient density in highly processed foods
Health implications	Nutrient deficiencies; relatively low chronic disease burden	Associations with obesity, cardiovascular disease, metabolic syndrome; emerging evidence for microbiome disruption and inflammation linked to additives
Diet–environment relationship	Strongly tied to local ecosystems and ecological availability	Increasingly detached from ecology; globalized, homogenized food supply
Evolutionary context	Continuity with ancestral diets; limited exposure to synthetic compounds	Introduction of compounds without evolutionary precedent, altering host–microbe interactions and long-term disease risk

To illustrate the contrast between ancestral and modern dietary patterns, this table summarizes key differences in food sources, processing techniques, nutritional quality, and ecological implications from pre and post-industrial diets. This comparison reinforces how industrial food practices differ from evolutionary dietary norms and raise new health and sustainability questions.

At a population level, post-industrial dietary changes enabled food security and accessibility, reducing the prevalence of deficiency-related diseases through practices such as fortification and enrichment. Yet they also narrowed dietary diversity, promoted energy-dense and nutrient-poor foods, and introduced synthetic molecules with no evolutionary precedent. The widespread availability of ultra-processed foods reflects the culmination of this historical process: a food environment where caloric sufficiency is abundant, but quality and evolutionary compatibility are increasingly compromised.

Beyond the shifts described in this review, it is likely that we are only beginning to understand the breadth of long-term health effects. Emerging concerns extend beyond food additives themselves to include compounds such as microplastics (50–53), which are not intentionally added but leach from packaging and containers into the food supply. These exposures highlight that the catalog of non-nutritive and synthetic dietary components is still expanding and that current evidence may represent only the “tip of the iceberg” with respect to their cumulative impacts on human physiology, microbiome function, and chronic disease risk.

Viewed from this perspective, the modern human diet is not simply the outcome of agricultural or industrial progress, but the result of a centuries-long layering of non-nutritive additives onto an ancestral nutritional template. This layering has contributed to dramatic shifts in metabolism, microbiome ecology, and chronic disease patterns. Recognizing this broad arc, from natural to synthetic, from ecological to industrial, provides a critical framework for re-evaluating dietary practices today.

Looking ahead to the coming years, we anticipate that the human diet will continue to evolve under the pressures of climate change, sustainability, technological innovation, and shifts in global food systems. Food and agriculture industries may incorporate more plant-forward, climate-resilient foods, lab-grown synthetic foods, and digitally optimized production systems (54). Together, these interactions may shape biological responses in ways that extend beyond classical nutrient sensing, as we see the increased prevalence of diseases such as metabolic syndrome, cardiovascular diseases, and cancer. We may see the evolution of future diets that emphasize nutritive composition (macro- and micronutrients) of food and their non-nutritive architecture (osmotic load, texture, and additive structure). This dual evolution underscores the need for broader mechanistic frameworks to anticipate how the diets of the future may interface with human physiology and health.

To address the question of this expanded mechanistic framework, we must first determine how non-nutritive dietary properties contribute to disease. Thus, future research may systematically dissect how factors such as osmolality, texture, and common additives influence gastrointestinal physiology. Experimental approaches that isolate these properties will be essential to define the molecular and cellular pathways through which they regulate functions in and out of the gut. Complementary observational, translational, and clinical studies should clarify the contribution of these non-nutritive properties to metabolic, inflammatory, and functional GI disorders. Prior research has investigated how food diversity shapes eating behavior. Likewise, it is vital to examine how food composition, especially non-nutritive components, affects physiological responses and the body’s overall interaction with food (55). Ultimately, identifying these mechanisms will be critical for predicting how increasingly engineered diets may shape disease risk and for guiding evidence-based dietary recommendations. By synthesizing findings from anthropology, genomics, ecology, and nutritional sciences, we can develop evolutionarily competent and sustainable dietary recommendations that facilitate a nuanced approach for a healthier future.
